# (*Z*)-2-(5-Fluoro-2-oxoindolin-3-yl­idene)-*N*-phenyl­hydrazinecarbothio­amide

**DOI:** 10.1107/S160053681105433X

**Published:** 2012-01-07

**Authors:** Amna Qasem Ali, Naser Eltaher Eltayeb, Siang Guan Teoh, Abdussalam Salhin, Hoong-Kun Fun

**Affiliations:** a School of Chemical Sciences, Universiti Sains Malaysia, Minden, Penang, Malaysia; b Faculty of Science, Sabha University, Libya; c Department of Chemistry, International University of Africa, Sudan; d X-ray Crystallography Unit, School of Physics,Universiti Sains Malaysia, 11800 USM, Penang, Malaysia

## Abstract

The title compound, C_15_H_11_FN_4_OS, crystallizes with three independent mol­ecules (*A*, *B* and *C*) in the asymmetric unit. The dihedral angles between the nine-membered 5-fluoro­indolin-2-one ring system and the benzene ring are 22.14 (11), 12.56 (11) and 3.70 (11)° in mol­ecules *A*, *B* and *C*, respectively. In all three mol­ecules, intra­molecular cyclic N—H⋯O and C—H⋯S hydrogen-bonding inter­actions [graph set *S*(6)] are present in the N—N—C—N chain between the ring systems. In the crystal, the *A* mol­ecules form centrosymmetric cyclic dimers through inter­molecular N—H⋯O hydrogen bonds, which are linked into a supramolecular chain along [100] via C—H⋯F interactions; each type of hydrogen bond has graph set graph set *R* ^2^_2_(8). A similar chain stabilised by similar interactions and also along [100] but, comprising alternating molecules of *B* and *C* is found. The latter chains are connected *via* C—H⋯S interactions, forming a layer with a zigzag topology parallel to (001).

## Related literature

For related structures, see: Qasem Ali *et al.* (2011*a* ▶,*b* ▶); Ferrari *et al.* (2002 ▶); Pervez *et al.* (2010 ▶); Ramzan *et al.* (2010 ▶). For the biological activity of Schiff bases, see: Bhandari *et al.* (2008 ▶); Bhardwaj *et al.* (2010 ▶); Pandeya *et al.* (1999 ▶); Sridhar *et al.* (2002 ▶); Suryavanshi & Pai (2006 ▶). For the cytotoxic and anti­cancer activity of isatin and its derivatives, see: Vine *et al.* (2009 ▶). For graph-set analysis, see Bernstein *et al.* (1995 ▶).
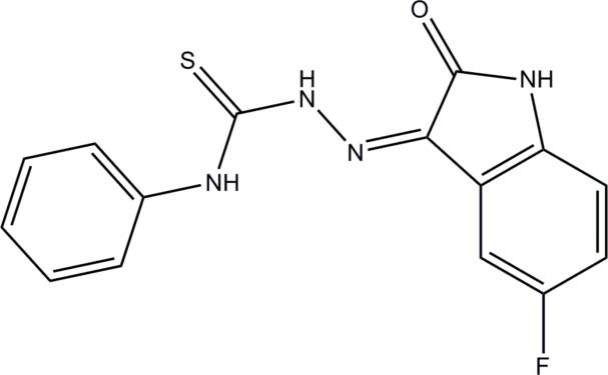


## Experimental

### 

#### Crystal data

C_15_H_11_FN_4_OS
*M* *_r_* = 314.34
Monoclinic, 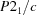

*a* = 14.8736 (7) Å
*b* = 17.4346 (9) Å
*c* = 18.1756 (10) Å
β = 116.023 (3)°
*V* = 4235.4 (4) Å^3^
*Z* = 12
Mo *K*α radiation
μ = 0.25 mm^−1^
*T* = 100 K
0.57 × 0.08 × 0.07 mm

#### Data collection

Bruker APEXII CCD diffractometer
Absorption correction: multi-scan (*SADABS*; Bruker, 2005 ▶) *T*_min_ = 0.872, *T*_max_ = 0.982
76994 measured reflections
9729 independent reflections
5962 reflections with *I* > 2σ(*I*)
*R*_int_ = 0.107

#### Refinement

*R*[*F* ^2^ > 2σ(*F* ^2^)] = 0.067
*wR*(*F* ^2^) = 0.128
*S* = 1.03
9729 reflections
595 parameters
H-atom parameters constrained
Δρ_max_ = 0.33 e Å^−3^
Δρ_min_ = −0.37 e Å^−3^

### 

Data collection: *APEX2* (Bruker, 2005 ▶); cell refinement: *SAINT* (Bruker, 2005 ▶); data reduction: *SAINT*; program(s) used to solve structure: *SHELXS97* (Sheldrick, 2008 ▶); program(s) used to refine structure: *SHELXL97* (Sheldrick, 2008 ▶); molecular graphics: *SHELXTL* (Sheldrick, 2008 ▶); software used to prepare material for publication: *SHELXTL* and *PLATON* (Spek, 2009 ▶).

## Supplementary Material

Crystal structure: contains datablock(s) I, global. DOI: 10.1107/S160053681105433X/zs2169sup1.cif

Structure factors: contains datablock(s) I. DOI: 10.1107/S160053681105433X/zs2169Isup2.hkl

Supplementary material file. DOI: 10.1107/S160053681105433X/zs2169Isup3.cml

Additional supplementary materials:  crystallographic information; 3D view; checkCIF report

## Figures and Tables

**Table 1:** Hydrogen-bond geometry (Å, °)

*D*—H⋯*A*	*D*—H	H⋯*A*	*D*⋯*A*	*D*—H⋯*A*
N1*A*—H10⋯O1*A*^i^	0.91	1.98	2.882 (3)	168
N3*A*—H11⋯O1*A*	0.81	2.10	2.752 (3)	138
N1*B*—H13⋯O1*C*^ii^	0.93	1.97	2.884 (3)	169
N3*B*—H14⋯O1*B*	0.76	2.16	2.773 (3)	138
N1*C*—H16⋯O1*B*^iii^	0.89	2.08	2.952 (3)	167
N3*C*—H17⋯O1*C*	0.85	2.07	2.729 (3)	134
C2*A*—H1⋯F1*A*^iv^	0.95	2.54	3.469 (4)	168
C2*B*—H4⋯F1*C*^v^	0.95	2.46	3.396 (4)	167
C11*A*—H11*A*⋯S1*A*	0.95	2.64	3.235 (3)	121
C11*B*—H11*B*⋯S1*B*	0.95	2.58	3.215 (3)	125
C11*C*—H11*C*⋯S1*C*	0.95	2.52	3.212 (3)	130
C13*B*—H13*B*⋯S1*C*	0.95	2.86	3.611 (3)	137

Symmetry codes: (i) 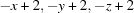; (ii) 
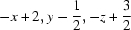
; (iii) 
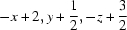
; (iv) 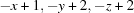; (v) 
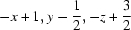
.

**Fig. 1.**
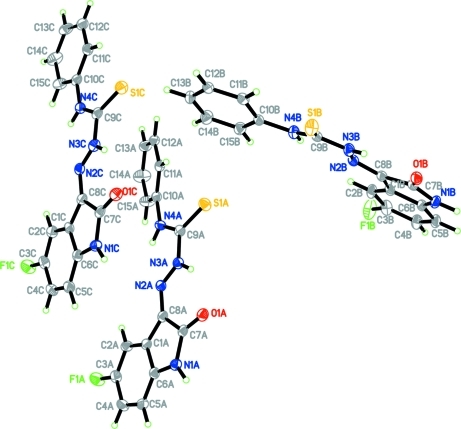
The molecular structure and the atom-numbering scheme for the three independent molecules of the title compound (A, B and C) in the asymmetric unit. Probability displacement ellipsoids are shown at the 50% level.

**Fig. 2.**
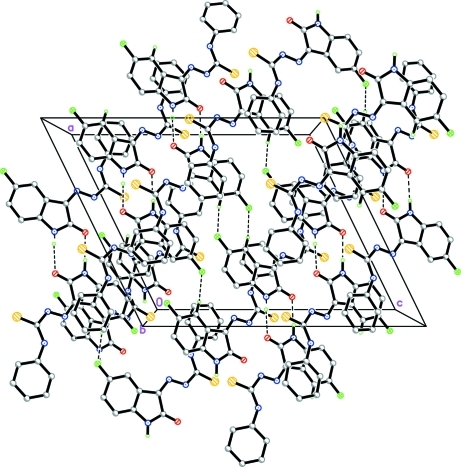
The crystal packing of the title compound viewed down the b axis. Hydrogen bonds are shown as dashed lines.

## supplementary crystallographic information

### Comment

Isatin (2,3-dioxindole) is an endogenous compound identified in humans, and its effect has been studied in a variety of systems (Vine *et al.*, 2009). Biological properties of isatin and its derivatives include a range of actions in the brain and offer protection against certain types of infections, such as anti-bacterial (Suryavanshi & Pai, 2006) antifungal, anticonvulsant, anti-HIV (Pandeya *et al.*, 1999), anti-depressant and anti-inflammatory activities (Bhandari *et al.*, 2008). In this paper we describe the single-crystal X-ray diffraction study of the title compound, C_15_H_11_FN_4_OS.

The title compound crystallizes with three independent molecules (*A*, *B* and *C*) in the asymmetric unit (Fig. 1). The dihedral angles between the nine-membered 5-fluoroindolin-2-one ring system and the benzene ring are 22.14 (11)°, 12.56 (11)° and 3.70 (11)° for molecules *A*, *B* and *C*, respectively. In molecule *A* intramolecular cyclic N3—H11···O1 and C11—H11···S1 hydrogen-bonding interactions [graph set *S*(6)] and N4—H12···N2 interactions [graph set *S*(5)] are present. In molecule *B*, intramolecular cyclic N3—H14···O1 and C11—H11···S1 hydrogen-bonding interactions [graph set *S*(6)] and N4—H15···N2 interactions [graph set *S*(5)] are present. In molecule *C* intramolecular cyclic N3—H17···O1 and C11—H11···S1 hydrogen-bonding interactions [graph set *S*(6)] and N4—H18···N2 interactions [graph set *S*(5)] are present (Bernstein *et al.*, 1995) (Table 1).

In the crystal the *A* molecules form centrosymmetric cyclic dimers through intermolecular C2—H1···F1 hydrogen bonds [graph set *R*^2^_2_(8)] and the *B* and *C* molecules are connected through intermolecular N1*B*—H13···O1*C*, N1*C*—H16··· O1*B*, C2*B*—H4···F1*C* and C13*B*—H13*B*··· S1*C* hydrogen bonds (Fig. 2). Weak C—H···π interactions are also present: C5*B*—H6···*Cg7*^vi^ = 3.583 (3) Å; C5*C*—H9 ···*Cg3*^vii^ = 3.496 (3)Å and C12*B*—H12*B*··· *Cg10*^viii^ = 3.738 (3)Å, where *Cg7*^vi^, *Cg3*^vii^, *Cg10*^viii^ are the centroids of the rings C10*A*—C15*A*, C10*B*—C15*B* and C1*C*— C6*C*, respectively [symmetry codes: (vi) -*x* + 2, *y* - 1/2, -*z* + 3/2; (vii)-*x* + 1, *y* + 1/2, -*z* + 2; (vii) -*x* + 1, -*y* + 2, -*z* + 1].

### Experimental

The Schiff base have been synthesized by refluxing the reaction mixture of an ethanolic solution (30 ml) of 4-phenyl-3-thiosemicarbazide (0.01 mol) and an ethanolic solution (30 ml) of 5-fluoroisatin (0.01 mol) for 2 hrs. The precipitate formed during reflux was filtered, washed with cold ethanol and recrystallized from hot ethanol. Yield (m.p.): 77% (517.4–518.0 K). The orange crystals were grown in acetone-DMF (3:1) by slow evaporation at room temperature.

### Refinement

The H atoms were positioned geometrically and refined using a riding model with C—H = 0.95 Å, N—H = 0.76–0.93 Å with *U*_iso_(H) = 1.2*U*_eq_(C) and 1.2*U*_eq_(N). The highest residual electron density peak (0.334 eÅ^-3^) is located at 0.86 Å from S1*C* and the deepest hole (-0.369 eÅ^-3^ is located at 0.79 Å from S1*C*.

### Crystal data

**Table:**

C_15_H_11_FN_4_OS	*F*(000) = 1944
*M**_r_* = 314.34	*D*_x_ = 1.479 Mg m^−^^3^
Monoclinic, *P*2_1_/*c*	Melting point = 517.4–518.0 K
Hall symbol: -P 2ybc	Mo *K*α radiation, λ = 0.71073 Å
*a* = 14.8736 (7) Å	Cell parameters from 8708 reflections
*b* = 17.4346 (9) Å	θ = 2.3–29.8°
*c* = 18.1756 (10) Å	µ = 0.25 mm^−^^1^
β = 116.023 (3)°	*T* = 100 K
*V* = 4235.4 (4) Å^3^	Needle, orange
*Z* = 12	0.57 × 0.08 × 0.07 mm

### Data collection

**Table:**

Bruker APEXII CCD diffractometer	9729 independent reflections
Radiation source: fine-focus sealed tube	5962 reflections with *I* > 2σ(*I*)
graphite	*R*_int_ = 0.107
φ and ω scans	θ_max_ = 27.5°, θ_min_ = 1.5°
Absorption correction: multi-scan (*SADABS*; Bruker, 2005)	*h* = −19→19
*T*_min_ = 0.872, *T*_max_ = 0.982	*k* = −22→22
76994 measured reflections	*l* = −23→23

### Refinement

**Table:**

Refinement on *F*^2^	Primary atom site location: structure-invariant direct methods
Least-squares matrix: full	Secondary atom site location: difference Fourier map
*R*[*F*^2^ > 2σ(*F*^2^)] = 0.067	Hydrogen site location: inferred from neighbouring sites
*wR*(*F*^2^) = 0.128	H-atom parameters constrained
*S* = 1.03	*w* = 1/[σ^2^(*F*_o_^2^) + (0.0374*P*)^2^ + 3.1211*P*] where *P* = (*F*_o_^2^ + 2*F*_c_^2^)/3
9729 reflections	(Δ/σ)_max_ = 0.001
595 parameters	Δρ_max_ = 0.33 e Å^−^^3^
0 restraints	Δρ_min_ = −0.37 e Å^−^^3^

### Special details

**Table:**

Geometry. All e.s.d.'s (except the e.s.d. in the dihedral angle between two l.s. planes) are estimated using the full covariance matrix. The cell e.s.d.'s are taken into account individually in the estimation of e.s.d.'s in distances, angles and torsion angles; correlations between e.s.d.'s in cell parameters are only used when they are defined by crystal symmetry. An approximate (isotropic) treatment of cell e.s.d.'s is used for estimating e.s.d.'s involving l.s. planes.
Refinement. Refinement of *F*^2^ against ALL reflections. The weighted *R*-factor *wR* and goodness of fit *S* are based on *F*^2^, conventional *R*-factors *R* are based on *F*, with *F* set to zero for negative *F*^2^. The threshold expression of *F*^2^ > σ(*F*^2^) is used only for calculating *R*-factors(gt) *etc*. and is not relevant to the choice of reflections for refinement. *R*-factors based on *F*^2^ are statistically about twice as large as those based on *F*, and *R*- factors based on ALL data will be even larger.

### Fractional atomic coordinates and isotropic or equivalent isotropic displacement parameters (Å^2^)

**Table:**

	*x*	*y*	*z*	*U*_iso_*/*U*_eq_	
S1A	0.69139 (5)	0.79737 (4)	0.70536 (5)	0.02538 (19)	
F1A	0.57560 (12)	1.08229 (9)	1.08785 (10)	0.0356 (4)	
O1A	0.89663 (13)	0.94925 (10)	0.91388 (11)	0.0226 (4)	
N1A	0.88969 (16)	1.02893 (12)	1.01394 (14)	0.0213 (5)	
H10	0.9555	1.0423	1.0389	0.026*	
N2A	0.67655 (16)	0.93040 (12)	0.87002 (14)	0.0195 (5)	
N3A	0.70701 (16)	0.89395 (12)	0.81983 (13)	0.0204 (5)	
H11	0.7654	0.8949	0.8299	0.024*	
N4A	0.54990 (16)	0.84497 (12)	0.75215 (13)	0.0195 (5)	
H12	0.5449	0.8685	0.7909	0.023*	
C1A	0.7264 (2)	1.01345 (14)	0.99082 (17)	0.0199 (6)	
C2A	0.6432 (2)	1.02354 (15)	1.00601 (17)	0.0240 (7)	
H1	0.5810	0.9996	0.9731	0.029*	
C3A	0.6557 (2)	1.07005 (15)	1.07123 (18)	0.0245 (7)	
C4A	0.7447 (2)	1.10575 (15)	1.12095 (18)	0.0282 (7)	
H2	0.7491	1.1371	1.1652	0.034*	
C5A	0.8282 (2)	1.09554 (15)	1.10586 (18)	0.0247 (7)	
H3	0.8901	1.1198	1.1388	0.030*	
C6A	0.8172 (2)	1.04888 (14)	1.04119 (17)	0.0208 (6)	
C7A	0.8516 (2)	0.98017 (14)	0.94941 (17)	0.0193 (6)	
C8A	0.7428 (2)	0.97019 (14)	0.92950 (17)	0.0195 (6)	
C9A	0.6439 (2)	0.84575 (14)	0.75900 (17)	0.0196 (6)	
C10A	0.4658 (2)	0.80134 (14)	0.69887 (17)	0.0201 (6)	
C11A	0.4509 (2)	0.77671 (14)	0.62183 (17)	0.0206 (6)	
H11A	0.4998	0.7870	0.6028	0.025*	
C12A	0.3644 (2)	0.73694 (14)	0.57258 (17)	0.0229 (7)	
H12A	0.3547	0.7198	0.5200	0.028*	
C13A	0.2928 (2)	0.72214 (16)	0.59901 (18)	0.0279 (7)	
H13A	0.2338	0.6949	0.5649	0.034*	
C14A	0.3072 (2)	0.74712 (19)	0.6757 (2)	0.0379 (8)	
H14A	0.2577	0.7371	0.6942	0.045*	
C15A	0.3932 (2)	0.78652 (17)	0.72557 (19)	0.0310 (7)	
H15A	0.4026	0.8035	0.7781	0.037*	
S1B	1.02133 (5)	0.71634 (4)	0.52383 (5)	0.02695 (19)	
F1B	0.92073 (13)	0.42288 (10)	0.90447 (12)	0.0436 (5)	
O1B	1.23203 (13)	0.55765 (10)	0.72239 (11)	0.0226 (5)	
N1B	1.23035 (16)	0.48096 (12)	0.82628 (14)	0.0214 (5)	
H13	1.2985	0.4702	0.8519	0.026*	
N2B	1.01071 (16)	0.57135 (12)	0.68099 (14)	0.0196 (5)	
N3B	1.03916 (17)	0.61167 (12)	0.63150 (14)	0.0209 (5)	
H14	1.0935	0.6109	0.6388	0.025*	
N4B	0.88131 (16)	0.65995 (12)	0.56800 (14)	0.0200 (5)	
H15	0.8715	0.6311	0.6040	0.024*	
C1B	1.0655 (2)	0.48839 (14)	0.80148 (17)	0.0196 (6)	
C2B	0.9833 (2)	0.47707 (15)	0.81741 (18)	0.0259 (7)	
H4	0.9192	0.4974	0.7829	0.031*	
C3B	1.0001 (2)	0.43443 (16)	0.88651 (19)	0.0280 (7)	
C4B	1.0909 (2)	0.40333 (15)	0.93804 (19)	0.0283 (7)	
H5	1.0977	0.3743	0.9844	0.034*	
C5B	1.1731 (2)	0.41475 (15)	0.92159 (18)	0.0254 (7)	
H6	1.2366	0.3934	0.9559	0.030*	
C6B	1.1593 (2)	0.45795 (14)	0.85392 (17)	0.0208 (6)	
C7B	1.1891 (2)	0.52651 (15)	0.75973 (17)	0.0208 (6)	
C8B	1.0795 (2)	0.53251 (14)	0.73940 (17)	0.0190 (6)	
C9B	0.9750 (2)	0.66235 (14)	0.57408 (16)	0.0203 (6)	
C10B	0.79530 (19)	0.70343 (14)	0.51813 (17)	0.0181 (6)	
C11B	0.7833 (2)	0.74138 (14)	0.44670 (16)	0.0204 (6)	
H11B	0.8348	0.7398	0.4292	0.024*	
C12B	0.6956 (2)	0.78141 (14)	0.40156 (17)	0.0225 (7)	
H12B	0.6878	0.8078	0.3534	0.027*	
C13B	0.6195 (2)	0.78350 (15)	0.42570 (18)	0.0252 (7)	
H13B	0.5598	0.8112	0.3945	0.030*	
C14B	0.6313 (2)	0.74470 (16)	0.49591 (18)	0.0283 (7)	
H14B	0.5789	0.7452	0.5125	0.034*	
C15B	0.7186 (2)	0.70523 (15)	0.54200 (18)	0.0242 (7)	
H15B	0.7262	0.6792	0.5903	0.029*	
S1C	0.36362 (5)	0.82275 (4)	0.38527 (5)	0.02614 (19)	
F1C	0.23568 (12)	1.07342 (9)	0.78204 (11)	0.0357 (4)	
O1C	0.55447 (13)	0.96915 (10)	0.59518 (12)	0.0236 (5)	
N1C	0.54937 (16)	1.03433 (12)	0.70483 (14)	0.0213 (5)	
H16	0.6130	1.0491	0.7283	0.026*	
N2C	0.33511 (16)	0.94551 (12)	0.55086 (14)	0.0204 (5)	
N3C	0.36712 (16)	0.91503 (12)	0.49839 (14)	0.0210 (5)	
H17	0.4266	0.9210	0.5046	0.025*	
N4C	0.21320 (16)	0.86212 (12)	0.42479 (13)	0.0195 (5)	
H18	0.2001	0.8867	0.4577	0.023*	
C1C	0.3849 (2)	1.01797 (14)	0.67892 (17)	0.0205 (6)	
C2C	0.3021 (2)	1.02535 (15)	0.69451 (18)	0.0238 (7)	
H7	0.2390	1.0043	0.6588	0.029*	
C3C	0.3159 (2)	1.06484 (15)	0.76469 (19)	0.0258 (7)	
C4C	0.4059 (2)	1.09627 (15)	0.81845 (19)	0.0278 (7)	
H8	0.4112	1.1228	0.8658	0.033*	
C5C	0.4885 (2)	1.08886 (15)	0.80277 (18)	0.0251 (7)	
H9	0.5513	1.1101	0.8388	0.030*	
C6C	0.4769 (2)	1.04974 (14)	0.73330 (18)	0.0210 (6)	
C7C	0.5098 (2)	0.99338 (15)	0.63443 (17)	0.0198 (6)	
C8C	0.4013 (2)	0.98208 (14)	0.61343 (17)	0.0202 (6)	
C9C	0.3080 (2)	0.86672 (14)	0.43534 (17)	0.0208 (6)	
C10C	0.1295 (2)	0.82099 (15)	0.36691 (17)	0.0212 (6)	
C11C	0.1298 (2)	0.78020 (15)	0.30086 (17)	0.0234 (7)	
H11C	0.1886	0.7781	0.2928	0.028*	
C12C	0.0435 (2)	0.74286 (15)	0.24716 (18)	0.0268 (7)	
H12C	0.0442	0.7146	0.2027	0.032*	
C13C	−0.0426 (2)	0.74592 (16)	0.25707 (18)	0.0304 (8)	
H13C	−0.1010	0.7197	0.2202	0.036*	
C14C	−0.0430 (2)	0.78793 (18)	0.32194 (19)	0.0344 (8)	
H14C	−0.1028	0.7914	0.3286	0.041*	
C15C	0.0423 (2)	0.82464 (17)	0.37664 (18)	0.0280 (7)	
H15C	0.0412	0.8525	0.4212	0.034*	

### Atomic displacement parameters (Å^2^)

**Table:**

	*U*^11^	*U*^22^	*U*^33^	*U*^12^	*U*^13^	*U*^23^
S1A	0.0234 (4)	0.0241 (4)	0.0304 (4)	−0.0009 (3)	0.0135 (4)	−0.0030 (3)
F1A	0.0313 (10)	0.0360 (10)	0.0440 (12)	−0.0058 (8)	0.0206 (9)	−0.0118 (9)
O1A	0.0184 (11)	0.0218 (10)	0.0250 (11)	0.0001 (8)	0.0072 (9)	0.0022 (9)
N1A	0.0158 (13)	0.0190 (11)	0.0250 (14)	−0.0037 (10)	0.0050 (11)	−0.0002 (11)
N2A	0.0208 (13)	0.0141 (11)	0.0213 (13)	−0.0006 (9)	0.0071 (11)	0.0014 (10)
N3A	0.0153 (13)	0.0200 (12)	0.0246 (14)	−0.0007 (10)	0.0074 (11)	−0.0008 (11)
N4A	0.0192 (13)	0.0189 (11)	0.0189 (13)	−0.0017 (9)	0.0071 (11)	−0.0034 (10)
C1A	0.0199 (16)	0.0148 (13)	0.0212 (16)	−0.0020 (11)	0.0054 (13)	0.0021 (12)
C2A	0.0236 (17)	0.0198 (14)	0.0235 (17)	−0.0046 (12)	0.0055 (14)	−0.0004 (13)
C3A	0.0230 (17)	0.0216 (14)	0.0305 (18)	−0.0003 (12)	0.0131 (15)	0.0005 (14)
C4A	0.0351 (19)	0.0191 (14)	0.0284 (18)	−0.0001 (13)	0.0122 (15)	−0.0030 (13)
C5A	0.0200 (17)	0.0217 (14)	0.0260 (17)	−0.0028 (12)	0.0042 (14)	−0.0024 (13)
C6A	0.0208 (16)	0.0152 (13)	0.0240 (16)	0.0003 (11)	0.0076 (13)	0.0032 (13)
C7A	0.0199 (16)	0.0136 (13)	0.0202 (16)	0.0003 (11)	0.0051 (13)	0.0070 (12)
C8A	0.0184 (16)	0.0154 (13)	0.0218 (16)	−0.0007 (11)	0.0062 (13)	0.0044 (12)
C9A	0.0218 (16)	0.0138 (13)	0.0219 (16)	−0.0029 (11)	0.0084 (13)	0.0023 (12)
C10A	0.0219 (16)	0.0159 (13)	0.0198 (16)	−0.0001 (11)	0.0067 (13)	0.0016 (12)
C11A	0.0200 (16)	0.0189 (14)	0.0224 (16)	0.0015 (12)	0.0088 (13)	0.0038 (13)
C12A	0.0271 (17)	0.0181 (14)	0.0189 (16)	0.0011 (12)	0.0057 (14)	0.0012 (12)
C13A	0.0239 (17)	0.0290 (16)	0.0260 (18)	−0.0042 (13)	0.0065 (14)	−0.0028 (14)
C14A	0.0256 (19)	0.055 (2)	0.037 (2)	−0.0165 (16)	0.0172 (16)	−0.0090 (18)
C15A	0.0273 (18)	0.0418 (18)	0.0266 (18)	−0.0103 (14)	0.0143 (15)	−0.0077 (15)
S1B	0.0224 (4)	0.0257 (4)	0.0346 (5)	0.0007 (3)	0.0141 (4)	0.0061 (4)
F1B	0.0325 (11)	0.0458 (11)	0.0595 (13)	0.0089 (9)	0.0265 (10)	0.0255 (10)
O1B	0.0182 (11)	0.0213 (10)	0.0269 (12)	0.0012 (8)	0.0085 (9)	−0.0022 (9)
N1B	0.0139 (12)	0.0201 (12)	0.0247 (14)	0.0013 (10)	0.0036 (11)	0.0004 (11)
N2B	0.0206 (13)	0.0145 (11)	0.0215 (13)	0.0007 (10)	0.0071 (11)	−0.0018 (10)
N3B	0.0165 (13)	0.0212 (12)	0.0259 (14)	0.0017 (10)	0.0101 (11)	−0.0002 (11)
N4B	0.0188 (13)	0.0180 (11)	0.0247 (14)	0.0011 (9)	0.0111 (11)	0.0033 (10)
C1B	0.0211 (16)	0.0100 (12)	0.0247 (16)	0.0005 (11)	0.0074 (13)	−0.0018 (12)
C2B	0.0217 (16)	0.0173 (14)	0.0340 (18)	0.0029 (12)	0.0079 (14)	0.0049 (14)
C3B	0.0254 (17)	0.0232 (15)	0.040 (2)	0.0007 (13)	0.0188 (16)	0.0053 (15)
C4B	0.0293 (18)	0.0207 (15)	0.0338 (19)	0.0051 (13)	0.0129 (16)	0.0085 (14)
C5B	0.0225 (17)	0.0176 (14)	0.0302 (18)	0.0054 (12)	0.0062 (14)	0.0033 (13)
C6B	0.0204 (16)	0.0130 (13)	0.0243 (17)	0.0019 (11)	0.0055 (13)	−0.0019 (12)
C7B	0.0204 (16)	0.0151 (13)	0.0238 (17)	0.0004 (12)	0.0068 (14)	−0.0073 (13)
C8B	0.0168 (15)	0.0146 (13)	0.0226 (16)	−0.0007 (11)	0.0060 (13)	−0.0045 (12)
C9B	0.0223 (16)	0.0145 (13)	0.0202 (16)	0.0017 (11)	0.0058 (13)	−0.0042 (12)
C10B	0.0174 (15)	0.0142 (13)	0.0197 (16)	0.0003 (11)	0.0055 (13)	−0.0023 (12)
C11B	0.0214 (16)	0.0185 (14)	0.0228 (16)	−0.0017 (12)	0.0110 (13)	−0.0029 (13)
C12B	0.0262 (17)	0.0167 (14)	0.0204 (16)	0.0017 (12)	0.0065 (14)	−0.0012 (12)
C13B	0.0213 (17)	0.0232 (15)	0.0265 (18)	0.0061 (12)	0.0062 (14)	−0.0009 (14)
C14B	0.0216 (17)	0.0361 (17)	0.0288 (18)	0.0044 (13)	0.0125 (15)	0.0018 (15)
C15B	0.0252 (17)	0.0253 (15)	0.0225 (17)	0.0026 (12)	0.0107 (14)	0.0022 (13)
S1C	0.0226 (4)	0.0296 (4)	0.0277 (4)	0.0005 (3)	0.0125 (4)	0.0002 (3)
F1C	0.0273 (10)	0.0381 (10)	0.0460 (12)	−0.0041 (8)	0.0200 (9)	−0.0108 (9)
O1C	0.0190 (11)	0.0264 (10)	0.0249 (12)	−0.0024 (8)	0.0093 (9)	0.0010 (9)
N1C	0.0142 (12)	0.0212 (12)	0.0243 (14)	−0.0044 (10)	0.0044 (11)	−0.0028 (11)
N2C	0.0199 (13)	0.0178 (11)	0.0217 (14)	0.0014 (10)	0.0075 (11)	0.0032 (11)
N3C	0.0150 (13)	0.0229 (12)	0.0241 (14)	−0.0028 (10)	0.0076 (11)	0.0019 (11)
N4C	0.0180 (13)	0.0181 (11)	0.0220 (13)	−0.0007 (9)	0.0083 (11)	0.0001 (10)
C1C	0.0236 (16)	0.0124 (13)	0.0234 (16)	−0.0002 (11)	0.0083 (14)	0.0010 (12)
C2C	0.0214 (16)	0.0204 (14)	0.0283 (18)	−0.0026 (12)	0.0096 (14)	0.0008 (13)
C3C	0.0249 (17)	0.0212 (15)	0.0347 (19)	0.0011 (12)	0.0162 (15)	−0.0004 (14)
C4C	0.0306 (18)	0.0204 (14)	0.0308 (19)	−0.0005 (13)	0.0118 (15)	−0.0038 (13)
C5C	0.0227 (17)	0.0199 (14)	0.0290 (18)	−0.0018 (12)	0.0080 (14)	−0.0017 (13)
C6C	0.0189 (16)	0.0148 (13)	0.0276 (17)	−0.0012 (11)	0.0086 (14)	0.0023 (13)
C7C	0.0192 (16)	0.0166 (13)	0.0200 (16)	−0.0006 (11)	0.0053 (13)	0.0060 (12)
C8C	0.0170 (15)	0.0156 (13)	0.0230 (16)	−0.0024 (11)	0.0040 (13)	0.0031 (13)
C9C	0.0228 (16)	0.0171 (13)	0.0190 (16)	−0.0010 (12)	0.0062 (13)	0.0063 (12)
C10C	0.0218 (16)	0.0171 (13)	0.0198 (16)	−0.0030 (12)	0.0046 (13)	0.0039 (12)
C11C	0.0228 (16)	0.0196 (14)	0.0261 (17)	0.0006 (12)	0.0091 (14)	0.0021 (13)
C12C	0.0294 (18)	0.0203 (14)	0.0235 (17)	−0.0022 (13)	0.0051 (14)	0.0017 (13)
C13C	0.0295 (19)	0.0301 (16)	0.0227 (17)	−0.0106 (14)	0.0032 (15)	0.0029 (14)
C14C	0.0221 (18)	0.050 (2)	0.0293 (19)	−0.0101 (15)	0.0101 (15)	0.0021 (17)
C15C	0.0253 (17)	0.0347 (17)	0.0235 (17)	−0.0068 (14)	0.0102 (14)	−0.0009 (14)

### Geometric parameters (Å, °)

**Table:**

S1A—C9A	1.663 (3)	C3B—C4B	1.373 (4)
F1A—C3A	1.368 (3)	C4B—C5B	1.394 (4)
O1A—C7A	1.239 (3)	C4B—H5	0.9500
N1A—C7A	1.355 (3)	C5B—C6B	1.379 (4)
N1A—C6A	1.414 (3)	C5B—H6	0.9500
N1A—H10	0.9100	C7B—C8B	1.509 (4)
N2A—C8A	1.298 (3)	C10B—C15B	1.387 (4)
N2A—N3A	1.343 (3)	C10B—C11B	1.397 (4)
N3A—C9A	1.378 (3)	C11B—C12B	1.386 (4)
N3A—H11	0.8057	C11B—H11B	0.9500
N4A—C9A	1.347 (3)	C12B—C13B	1.383 (4)
N4A—C10A	1.420 (3)	C12B—H12B	0.9500
N4A—H12	0.8455	C13B—C14B	1.386 (4)
C1A—C2A	1.391 (4)	C13B—H13B	0.9500
C1A—C6A	1.400 (4)	C14B—C15B	1.382 (4)
C1A—C8A	1.452 (4)	C14B—H14B	0.9500
C2A—C3A	1.380 (4)	C15B—H15B	0.9500
C2A—H1	0.9500	S1C—C9C	1.661 (3)
C3A—C4A	1.381 (4)	F1C—C3C	1.369 (3)
C4A—C5A	1.395 (4)	O1C—C7C	1.243 (3)
C4A—H2	0.9500	N1C—C7C	1.354 (3)
C5A—C6A	1.379 (4)	N1C—C6C	1.411 (3)
C5A—H3	0.9500	N1C—H16	0.8888
C7A—C8A	1.505 (4)	N2C—C8C	1.299 (3)
C10A—C11A	1.386 (4)	N2C—N3C	1.348 (3)
C10A—C15A	1.389 (4)	N3C—C9C	1.382 (3)
C11A—C12A	1.388 (4)	N3C—H17	0.8480
C11A—H11A	0.9500	N4C—C9C	1.340 (3)
C12A—C13A	1.371 (4)	N4C—C10C	1.422 (3)
C12A—H12A	0.9500	N4C—H18	0.8265
C13A—C14A	1.385 (4)	C1C—C2C	1.385 (4)
C13A—H13A	0.9500	C1C—C6C	1.404 (4)
C14A—C15A	1.383 (4)	C1C—C8C	1.457 (4)
C14A—H14A	0.9500	C2C—C3C	1.384 (4)
C15A—H15A	0.9500	C2C—H7	0.9500
S1B—C9B	1.657 (3)	C3C—C4C	1.378 (4)
F1B—C3B	1.371 (3)	C4C—C5C	1.384 (4)
O1B—C7B	1.242 (3)	C4C—H8	0.9500
N1B—C7B	1.349 (3)	C5C—C6C	1.378 (4)
N1B—C6B	1.413 (3)	C5C—H9	0.9500
N1B—H13	0.9300	C7C—C8C	1.500 (4)
N2B—C8B	1.296 (3)	C10C—C15C	1.386 (4)
N2B—N3B	1.348 (3)	C10C—C11C	1.397 (4)
N3B—C9B	1.381 (3)	C11C—C12C	1.387 (4)
N3B—H14	0.7595	C11C—H11C	0.9500
N4B—C9B	1.349 (3)	C12C—C13C	1.370 (4)
N4B—C10B	1.420 (3)	C12C—H12C	0.9500
N4B—H15	0.8864	C13C—C14C	1.391 (4)
C1B—C2B	1.388 (4)	C13C—H13C	0.9500
C1B—C6B	1.404 (4)	C14C—C15C	1.379 (4)
C1B—C8B	1.455 (4)	C14C—H14C	0.9500
C2B—C3B	1.384 (4)	C15C—H15C	0.9500
C2B—H4	0.9500		
			
C7A—N1A—C6A	111.3 (2)	O1B—C7B—N1B	127.4 (3)
C7A—N1A—H10	122.8	O1B—C7B—C8B	126.5 (3)
C6A—N1A—H10	125.5	N1B—C7B—C8B	106.1 (2)
C8A—N2A—N3A	117.4 (2)	N2B—C8B—C1B	125.5 (2)
N2A—N3A—C9A	121.3 (2)	N2B—C8B—C7B	128.1 (3)
N2A—N3A—H11	119.2	C1B—C8B—C7B	106.3 (2)
C9A—N3A—H11	118.8	N4B—C9B—N3B	112.9 (2)
C9A—N4A—C10A	129.4 (2)	N4B—C9B—S1B	129.5 (2)
C9A—N4A—H12	112.6	N3B—C9B—S1B	117.6 (2)
C10A—N4A—H12	117.1	C15B—C10B—C11B	119.5 (2)
C2A—C1A—C6A	120.1 (3)	C15B—C10B—N4B	116.5 (2)
C2A—C1A—C8A	132.9 (2)	C11B—C10B—N4B	123.9 (2)
C6A—C1A—C8A	107.0 (2)	C12B—C11B—C10B	119.5 (3)
C3A—C2A—C1A	116.6 (3)	C12B—C11B—H11B	120.3
C3A—C2A—H1	121.7	C10B—C11B—H11B	120.3
C1A—C2A—H1	121.7	C13B—C12B—C11B	120.9 (3)
F1A—C3A—C2A	118.6 (2)	C13B—C12B—H12B	119.5
F1A—C3A—C4A	117.6 (3)	C11B—C12B—H12B	119.5
C2A—C3A—C4A	123.8 (3)	C12B—C13B—C14B	119.3 (3)
C3A—C4A—C5A	119.6 (3)	C12B—C13B—H13B	120.4
C3A—C4A—H2	120.2	C14B—C13B—H13B	120.4
C5A—C4A—H2	120.2	C15B—C14B—C13B	120.4 (3)
C6A—C5A—C4A	117.4 (3)	C15B—C14B—H14B	119.8
C6A—C5A—H3	121.3	C13B—C14B—H14B	119.8
C4A—C5A—H3	121.3	C14B—C15B—C10B	120.4 (3)
C5A—C6A—C1A	122.5 (3)	C14B—C15B—H15B	119.8
C5A—C6A—N1A	128.3 (3)	C10B—C15B—H15B	119.8
C1A—C6A—N1A	109.1 (2)	C7C—N1C—C6C	111.2 (2)
O1A—C7A—N1A	127.4 (3)	C7C—N1C—H16	122.4
O1A—C7A—C8A	126.5 (2)	C6C—N1C—H16	126.4
N1A—C7A—C8A	106.1 (2)	C8C—N2C—N3C	116.5 (2)
N2A—C8A—C1A	126.5 (2)	N2C—N3C—C9C	122.2 (2)
N2A—C8A—C7A	127.2 (3)	N2C—N3C—H17	121.9
C1A—C8A—C7A	106.3 (2)	C9C—N3C—H17	115.6
N4A—C9A—N3A	113.2 (2)	C9C—N4C—C10C	131.4 (2)
N4A—C9A—S1A	129.1 (2)	C9C—N4C—H18	115.7
N3A—C9A—S1A	117.6 (2)	C10C—N4C—H18	112.9
C11A—C10A—C15A	119.4 (3)	C2C—C1C—C6C	119.9 (3)
C11A—C10A—N4A	123.9 (2)	C2C—C1C—C8C	133.6 (3)
C15A—C10A—N4A	116.6 (3)	C6C—C1C—C8C	106.5 (2)
C10A—C11A—C12A	119.9 (3)	C3C—C2C—C1C	116.7 (3)
C10A—C11A—H11A	120.0	C3C—C2C—H7	121.6
C12A—C11A—H11A	120.0	C1C—C2C—H7	121.6
C13A—C12A—C11A	120.7 (3)	F1C—C3C—C4C	117.8 (3)
C13A—C12A—H12A	119.7	F1C—C3C—C2C	118.4 (3)
C11A—C12A—H12A	119.7	C4C—C3C—C2C	123.8 (3)
C12A—C13A—C14A	119.5 (3)	C3C—C4C—C5C	119.4 (3)
C12A—C13A—H13A	120.2	C3C—C4C—H8	120.3
C14A—C13A—H13A	120.2	C5C—C4C—H8	120.3
C15A—C14A—C13A	120.4 (3)	C6C—C5C—C4C	118.0 (3)
C15A—C14A—H14A	119.8	C6C—C5C—H9	121.0
C13A—C14A—H14A	119.8	C4C—C5C—H9	121.0
C14A—C15A—C10A	120.1 (3)	C5C—C6C—C1C	122.2 (3)
C14A—C15A—H15A	120.0	C5C—C6C—N1C	128.4 (2)
C10A—C15A—H15A	120.0	C1C—C6C—N1C	109.4 (2)
C7B—N1B—C6B	111.7 (2)	O1C—C7C—N1C	127.0 (3)
C7B—N1B—H13	122.3	O1C—C7C—C8C	126.6 (3)
C6B—N1B—H13	125.8	N1C—C7C—C8C	106.4 (2)
C8B—N2B—N3B	116.9 (2)	N2C—C8C—C1C	126.3 (3)
N2B—N3B—C9B	121.5 (2)	N2C—C8C—C7C	127.2 (3)
N2B—N3B—H14	119.7	C1C—C8C—C7C	106.4 (2)
C9B—N3B—H14	118.2	N4C—C9C—N3C	113.7 (2)
C9B—N4B—C10B	130.1 (2)	N4C—C9C—S1C	129.8 (2)
C9B—N4B—H15	117.1	N3C—C9C—S1C	116.6 (2)
C10B—N4B—H15	112.3	C15C—C10C—C11C	119.3 (3)
C2B—C1B—C6B	120.6 (3)	C15C—C10C—N4C	116.6 (3)
C2B—C1B—C8B	132.6 (3)	C11C—C10C—N4C	124.2 (3)
C6B—C1B—C8B	106.7 (2)	C12C—C11C—C10C	119.6 (3)
C3B—C2B—C1B	116.1 (3)	C12C—C11C—H11C	120.2
C3B—C2B—H4	121.9	C10C—C11C—H11C	120.2
C1B—C2B—H4	121.9	C13C—C12C—C11C	121.2 (3)
F1B—C3B—C4B	118.1 (3)	C13C—C12C—H12C	119.4
F1B—C3B—C2B	117.6 (3)	C11C—C12C—H12C	119.4
C4B—C3B—C2B	124.3 (3)	C12C—C13C—C14C	119.0 (3)
C3B—C4B—C5B	119.3 (3)	C12C—C13C—H13C	120.5
C3B—C4B—H5	120.3	C14C—C13C—H13C	120.5
C5B—C4B—H5	120.3	C15C—C14C—C13C	120.7 (3)
C6B—C5B—C4B	117.9 (3)	C15C—C14C—H14C	119.7
C6B—C5B—H6	121.0	C13C—C14C—H14C	119.7
C4B—C5B—H6	121.0	C14C—C15C—C10C	120.2 (3)
C5B—C6B—C1B	121.8 (3)	C14C—C15C—H15C	119.9
C5B—C6B—N1B	129.1 (2)	C10C—C15C—H15C	119.9
C1B—C6B—N1B	109.1 (2)		
			
C8A—N2A—N3A—C9A	175.0 (2)	C6B—C1B—C8B—N2B	−178.0 (2)
C6A—C1A—C2A—C3A	0.8 (4)	C2B—C1B—C8B—C7B	175.3 (3)
C8A—C1A—C2A—C3A	−179.8 (3)	C6B—C1B—C8B—C7B	−0.6 (3)
C1A—C2A—C3A—F1A	179.3 (2)	O1B—C7B—C8B—N2B	−1.0 (4)
C1A—C2A—C3A—C4A	−0.3 (4)	N1B—C7B—C8B—N2B	178.8 (2)
F1A—C3A—C4A—C5A	−179.4 (2)	O1B—C7B—C8B—C1B	−178.3 (2)
C2A—C3A—C4A—C5A	0.2 (4)	N1B—C7B—C8B—C1B	1.5 (3)
C3A—C4A—C5A—C6A	−0.5 (4)	C10B—N4B—C9B—N3B	178.6 (2)
C4A—C5A—C6A—C1A	1.0 (4)	C10B—N4B—C9B—S1B	−1.2 (4)
C4A—C5A—C6A—N1A	−179.4 (3)	N2B—N3B—C9B—N4B	−6.7 (3)
C2A—C1A—C6A—C5A	−1.2 (4)	N2B—N3B—C9B—S1B	173.10 (18)
C8A—C1A—C6A—C5A	179.3 (2)	C9B—N4B—C10B—C15B	−161.1 (3)
C2A—C1A—C6A—N1A	179.1 (2)	C9B—N4B—C10B—C11B	21.0 (4)
C8A—C1A—C6A—N1A	−0.4 (3)	C15B—C10B—C11B—C12B	1.1 (4)
C7A—N1A—C6A—C5A	178.6 (3)	N4B—C10B—C11B—C12B	179.0 (2)
C7A—N1A—C6A—C1A	−1.8 (3)	C10B—C11B—C12B—C13B	−0.8 (4)
C6A—N1A—C7A—O1A	−176.4 (2)	C11B—C12B—C13B—C14B	−0.2 (4)
C6A—N1A—C7A—C8A	3.1 (3)	C12B—C13B—C14B—C15B	0.9 (4)
N3A—N2A—C8A—C1A	−179.6 (2)	C13B—C14B—C15B—C10B	−0.6 (4)
N3A—N2A—C8A—C7A	−0.3 (4)	C11B—C10B—C15B—C14B	−0.5 (4)
C2A—C1A—C8A—N2A	2.2 (5)	N4B—C10B—C15B—C14B	−178.5 (2)
C6A—C1A—C8A—N2A	−178.4 (2)	C8C—N2C—N3C—C9C	171.6 (2)
C2A—C1A—C8A—C7A	−177.3 (3)	C6C—C1C—C2C—C3C	0.1 (4)
C6A—C1A—C8A—C7A	2.2 (3)	C8C—C1C—C2C—C3C	−179.6 (3)
O1A—C7A—C8A—N2A	−3.1 (4)	C1C—C2C—C3C—F1C	179.9 (2)
N1A—C7A—C8A—N2A	177.3 (2)	C1C—C2C—C3C—C4C	−0.1 (4)
O1A—C7A—C8A—C1A	176.3 (2)	F1C—C3C—C4C—C5C	−179.9 (2)
N1A—C7A—C8A—C1A	−3.2 (3)	C2C—C3C—C4C—C5C	0.1 (4)
C10A—N4A—C9A—N3A	−178.0 (2)	C3C—C4C—C5C—C6C	−0.1 (4)
C10A—N4A—C9A—S1A	1.8 (4)	C4C—C5C—C6C—C1C	0.2 (4)
N2A—N3A—C9A—N4A	5.1 (3)	C4C—C5C—C6C—N1C	−179.5 (3)
N2A—N3A—C9A—S1A	−174.76 (18)	C2C—C1C—C6C—C5C	−0.2 (4)
C9A—N4A—C10A—C11A	−29.5 (4)	C8C—C1C—C6C—C5C	179.6 (2)
C9A—N4A—C10A—C15A	153.7 (3)	C2C—C1C—C6C—N1C	179.6 (2)
C15A—C10A—C11A—C12A	−0.7 (4)	C8C—C1C—C6C—N1C	−0.7 (3)
N4A—C10A—C11A—C12A	−177.4 (2)	C7C—N1C—C6C—C5C	179.3 (3)
C10A—C11A—C12A—C13A	0.5 (4)	C7C—N1C—C6C—C1C	−0.4 (3)
C11A—C12A—C13A—C14A	−0.1 (4)	C6C—N1C—C7C—O1C	−178.1 (2)
C12A—C13A—C14A—C15A	−0.2 (5)	C6C—N1C—C7C—C8C	1.3 (3)
C13A—C14A—C15A—C10A	0.0 (5)	N3C—N2C—C8C—C1C	−178.4 (2)
C11A—C10A—C15A—C14A	0.4 (4)	N3C—N2C—C8C—C7C	−0.8 (4)
N4A—C10A—C15A—C14A	177.3 (3)	C2C—C1C—C8C—N2C	−0.9 (5)
C8B—N2B—N3B—C9B	−171.8 (2)	C6C—C1C—C8C—N2C	179.4 (2)
C6B—C1B—C2B—C3B	−0.4 (4)	C2C—C1C—C8C—C7C	−178.9 (3)
C8B—C1B—C2B—C3B	−175.9 (3)	C6C—C1C—C8C—C7C	1.4 (3)
C1B—C2B—C3B—F1B	179.3 (2)	O1C—C7C—C8C—N2C	−0.3 (4)
C1B—C2B—C3B—C4B	−0.5 (4)	N1C—C7C—C8C—N2C	−179.6 (2)
F1B—C3B—C4B—C5B	−179.5 (2)	O1C—C7C—C8C—C1C	177.7 (2)
C2B—C3B—C4B—C5B	0.3 (5)	N1C—C7C—C8C—C1C	−1.6 (3)
C3B—C4B—C5B—C6B	0.6 (4)	C10C—N4C—C9C—N3C	178.6 (2)
C4B—C5B—C6B—C1B	−1.5 (4)	C10C—N4C—C9C—S1C	−2.3 (4)
C4B—C5B—C6B—N1B	176.6 (3)	N2C—N3C—C9C—N4C	8.8 (3)
C2B—C1B—C6B—C5B	1.4 (4)	N2C—N3C—C9C—S1C	−170.47 (18)
C8B—C1B—C6B—C5B	177.9 (2)	C9C—N4C—C10C—C15C	175.6 (3)
C2B—C1B—C6B—N1B	−177.0 (2)	C9C—N4C—C10C—C11C	−6.5 (4)
C8B—C1B—C6B—N1B	−0.5 (3)	C15C—C10C—C11C—C12C	−1.3 (4)
C7B—N1B—C6B—C5B	−176.7 (3)	N4C—C10C—C11C—C12C	−179.1 (2)
C7B—N1B—C6B—C1B	1.6 (3)	C10C—C11C—C12C—C13C	0.8 (4)
C6B—N1B—C7B—O1B	177.9 (2)	C11C—C12C—C13C—C14C	0.6 (4)
C6B—N1B—C7B—C8B	−1.9 (3)	C12C—C13C—C14C—C15C	−1.4 (4)
N3B—N2B—C8B—C1B	176.9 (2)	C13C—C14C—C15C—C10C	0.9 (5)
N3B—N2B—C8B—C7B	0.1 (4)	C11C—C10C—C15C—C14C	0.4 (4)
C2B—C1B—C8B—N2B	−2.0 (5)	N4C—C10C—C15C—C14C	178.4 (3)

### Hydrogen-bond geometry (Å, °)

**Table:**

*D*—H···*A*	*D*—H	H···*A*	*D*···*A*	*D*—H···*A*
N1A—H10···O1A^i^	0.91	1.98	2.882 (3)	168.
N3A—H11···O1A	0.81	2.10	2.752 (3)	138.
N4A—H12···N2A	0.85	2.14	2.611 (3)	114.
N1B—H13···O1C^ii^	0.93	1.97	2.884 (3)	169.
N3B—H14···O1B	0.76	2.16	2.773 (3)	138.
N4B—H15···N2B	0.89	2.19	2.615 (3)	109.
N1C—H16···O1B^iii^	0.89	2.08	2.952 (3)	167.
N3C—H17···O1C	0.85	2.07	2.729 (3)	134.
N4C—H18···N2C	0.83	2.23	2.647 (3)	112.
C2A—H1···F1A^iv^	0.95	2.54	3.469 (4)	168.
C2B—H4···F1C^v^	0.95	2.46	3.396 (4)	167.
C11A—H11A···S1A	0.95	2.64	3.235 (3)	121.
C11B—H11B···S1B	0.95	2.58	3.215 (3)	125.
C11C—H11C···S1C	0.95	2.52	3.212 (3)	130.
C13B—H13B···S1C	0.95	2.86	3.611 (3)	137.

Symmetry codes: (i) −*x*+2, −*y*+2, −*z*+2; (ii) −*x*+2, *y*−1/2, −*z*+3/2; (iii) −*x*+2, *y*+1/2, −*z*+3/2; (iv) −*x*+1, −*y*+2, −*z*+2; (v) −*x*+1, *y*−1/2, −*z*+3/2.
